# Postural Difference between the Interventions Reflecting the Concept of Mirror Therapy in Healthy Subjects

**DOI:** 10.3390/brainsci11121567

**Published:** 2021-11-27

**Authors:** Jinmin Kim, Changho Song

**Affiliations:** 1 School of Sport, Exercise and Rehabilitation Sciences, University of Birmingham, Edgbaston, Birmingham B15 2TT, UK; 36jinmin@gmail.com; 2 Department of Physical Therapy, College of Health and Welfare, Sahmyook University, Seoul 01795, Korea

**Keywords:** mirror therapy, virtual reality, posture, kinematic analysis

## Abstract

(1) Background: Mirror therapy is one of the promising interventions for the upper limb rehabilitation of stroke patients. Postural asymmetry during mirror therapy was pointed out as a possibility to influence stroke patients’ rehabilitation negatively. However, it is still difficult to find studies on the postural changes in mirror therapy concept interventions. This study compared three methods of postural differences as follows: traditional mirror therapy (mirror); displaying the real-time movement of the unaffected side on the screen above the affected side (screen); and playing a pre-recorded movement of the unaffected side on a tablet placed on a movable box where the affected hand is put inside (movable). (2) Methods: to observe a kinematic difference, we recruited 16 healthy volunteers to go through three different interventions (mirror, screen, movable). The motion capture system made observations on the postures before and during interventions, then compared and analyzed. (3) Results: while using the mirror, the sitting posture was observed to become asymmetric, and the following unique posture was observed where the target hand went further from the trunk while performing tasks. In addition, the shoulder of the target side came forward, and the difference between both elbow flexion angles was also observed. On the other hand, the screen or movable device did not cause a significant change in the sitting posture, and no additional postural differences were observed either. (4) Conclusions: mirror therapy showed a tendency to cause lateral flexion opposite the target hand, thus, creating additional postural change. However, developed methods controlled spine tilt, and enabled the keeping of the midline while sitting during the intervention.

## 1. Introduction

Stroke is one of the leading causes of long-term disability, occurring in many patients annually [1]. Hemiparesis is one of the phenotypes of a stroke, which decreases the use of the affected limbs and, in many cases, decreases the weight-bearing of the affected side [2]. Therefore, midline orientation training precedes the rehabilitation of hemiparetic patients. One of the essential steps in helping stroke patients return to daily life is keeping the midline while controlling their proximal part and performing tasks using their distal part [3].

One of the most challenging parts of stroke rehabilitation is in the upper limb. Upper limb rehabilitation is complex because many cases fail to reach the aimed goal in the distal part of the upper limb, where massive fine movements occur. Therefore, rehabilitation of the upper limb is challenging for both physiotherapists and stroke survivors [4]. 

Mirror therapy is one of the promising interventions for upper limb rehabilitation for stroke survivors. Mirror therapy was first proposed to relieve phantom limb pain in amputees [5]. In mirror therapy, the mirror is not placed facing the patient, but between the two arms where the reflective side faces one arm, and the other side is hidden behind the mirror. After observing amputees, mirror therapy was soon suggested for the upper limb rehabilitation of stroke patients [6]. This cost-effective and patient-led approach drew interest from many stroke patients and clinical researchers. Numerous studies were carried out on stroke patients, and it was concluded that it was effective in improving motor function [7]. Positive results were reported in all acute, subacute, and chronic patients [8]. 

However, a few issues were raised for the methodology of mirror therapy [7]. It was pointed out that hiding the affected hand behind the mirror and tilting the trunk to the unaffected side during an intervention may cause problems [9]. Methods such as putting a big mirror up to eye level, and using advanced technology were suggested to prevent sitting posture asymmetry during mirror therapy [10,11]. 

Placing a screen on the affected limb was suggested to decrease the lateral tilt, and induce visual illusion. In, Jung, Lee, and Song [10] applied a “virtual reality reflection device” based on the concept of mirror therapy. In and his colleagues placed a monitor over the affected hand which showed the captured real-time image of the unaffected limb. A webcam was used to capture the unaffected limb, and then the image was flipped mirror-symmetrically, and shown on the monitor as if looking at the affected hand. 

Lee, Lee, Lee, and Song [11] also placed a monitor over the affected limb. However, they recorded the motions of the unaffected limb for the task before the intervention. Then, the video was shown on the screen as the actual asymmetric timing intervention. Lee and his colleagues provide ideas that could help develop movable or wearable devices that give the illusion on the target side. The method that puts a screen over the target arm and provides an illusion along with the arm motion shows the direction of where a possible future movable or wearable device can go. Such a novel method may help users use only the target arm and enjoy the illusion in a preferred posture, not requiring bimanual movements during the intervention. 

Many mirror therapy methods using advanced apparatus can be suggested to resolve sitting asymmetry. However, studies on observing the differences between kinematic variables are scarce. 

Therefore, our kinematic study is to observe the difference in the postural alignment shown while using the various types of illusive intervention (mirror, real-time screen device, movable screen device), and investigate positional changes in the trunk.

## 2. Materials and Methods

### 2.1. Participants

Sixteen (eight males, eight females) healthy undergraduate students (mean age: 22.9 ± 2.3) volunteered for this experiment. All participants were right-handed, confirmed with Edinburgh handedness inventory (EHI) (Oldfield, 1971) (mean score: 92.5 ± 9.8). They were all students who were naïve to the mirror therapy. Participants did not have musculoskeletal or neurological problems that could affect the experiment, and they had no visual impairment. This study was approved by the Sahmyook University Ethics Committee(2-1040781-AB-N-01-2017074HR), and participants provided written informed consent prior to taking part. 

### 2.2. Apparatus

The experiment was carried out in a laboratory set up with a motion capture system. The room was restricted for the participant to have undivided attention during the task. All unnecessary objects were removed from the range of eyesight during the trial. 

Thirteen infrared cameras (Miqus, Qualisys Ltd., Göteborg, Sweden) were connected to a motion capture system (Qualisys Track Manager, Qualisys Ltd., Göteborg, Sweden) to capture the motion. The sampling rate of the motion capture camera was set to 200 Hz. Subjects wore a prepared top and bottom suit to attach 14 mm reflective spherical markers, and the markers were attached to the following position: posterior head; C7; T3; T7; T12; L5; sacrum; left acromion; right acromion; left elbow; right elbow; left wrist; right wrist (13 in total). 

The mirror (width 45 cm × height 55 cm) was positioned on a table, midline to the subject. The mirror was fixated with wooden blocks. Participants had their hands on the table comfortably, placing them at the same distance from the mirror.

A 12.9-inch display tablet (12.9-inch iPad Pro, Apple Inc., Cupertino, CA, USA) was used for the screen device. The screen device was used to provide an illusion like a mirror. In place of the screen in In et al., an iPad was used, and a secure iPad mount for the desk was used to fix the pad above the hands. The screen was to show the movement of the opposite hand. Thus, a smartphone was used to send the real-time movement of the opposite hand to the screen. Before sending to the screen, the captured image was flipped mirror symmetrically, and the initial posture of the user was equivalent to that of the posture in the mirror condition. The size of the displayed arm was similar to the real arm placed below the tablet.

**Figure 1:**
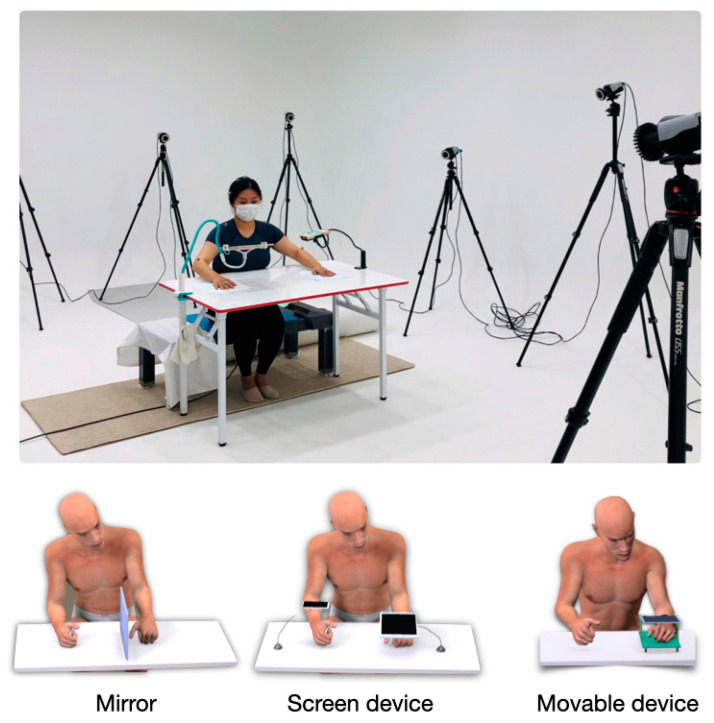
The top image demonstrates the experimental environment. Images below show the devices used for the three different conditions (mirror, screen, movable).

In the movable condition, the same 12.9-inch display tablet was used. The tablet was then placed on top of a transparent plastic box. The target arm was placed in the plastic box, and the video playing on the screen was the pre-recorded movements of the opposite arm. The pre-recorded video was flipped mirror-symmetrically to give the illusion that it was the hand in the movable device. Since the movable device only needs the target arm, the other arm was placed to hold the edge of the desk so that it does not interfere with the workspace. As the movable condition is not a bimanual task, participants were asked to begin it in their most comfortable and preferable position (Figure 1).

### 2.3. Task, Design, and Procedure

The clench-and-unfold task was instructed to fix the forearm and minimize postural changes during the task. Both arms were to be placed on the table shoulder-length wide, with the same distance from the mirror. Before the experiment, a metronome was used for practice in performing one task per second. Each trial lasted for 15 s. Participants repeated the task five times during each condition, and the order of the condition was randomized. The six conditions were as follows: right mirror when the head is tilted to left and face the right; left mirror when the head is tilted to the right and face the left; right screen when the head is turned right to face the screen; left screen when the head is turned left to face the screen; right movable when the right hand used the movable device; left movable when the left hand used the movable device. A total of 30 trials were carried out in a total of six conditions. Participants were always asked to gaze at the hand shown on the screen or mirror during the intervention. 

Prior to the experiment, participants were informed of the procedure, and if they had any questions, they were answered. Then, they signed the written consent form. 

In each trial, there were three verbal orders (“ready”, “go”, “stop”). The experiment commenced with a “ready” signal, and the participant sat looking forward while keeping the midline by their judgment. At the “go” signal, the participant began the task in the given condition. After 15 s, the “stop” sign was called, and the participant returned to the resting position. Between the trials, there was a 10-s break to go on to the following trial condition. After ten trials, there was a 5-min scheduled break. Other than this, at the participant’s request, sufficient break time was allowed. 

### 2.4. Data Analysis

The coordinates of markers received by the motion capture system were exported to the Matlab file, then analyzed by Matlab (Matlab 2021a, Mathworks Inc., Natick, MA, USA). Signals were rectified and filtered in with a low-pass Butterworth filter from motion capture software. The main variables were: flexion and lateral flexion of the spinal segments; elbow flexion angle difference between both arms; and shoulder angle difference in the horizontal plane. Once the task begins, the participants keep the posture until the end. The initial posture at the “ready” signal and the intervention posture during the task after the “go” signal were compared to check how the posture changes. The definition of *initial posture* in this study is the average coordinate value of the posture kept for 0.5 s after the “ready” signal. The definition of intervention posture in this study is the average coordinate value of the posture kept for 0.5 s two seconds before the end of the task. These postures were then compared with the preparing posture. 

Prior to observing the spinal tilt, six spinal segments were defined. Segments are as follows: posterior head to C7; C7 to T3; T3 to T7; T7 to T12; T12 to L5; L5 to the sacrum. We compared the difference between the segment’s initial posture and intervention posture showed in each condition. Lateral tilt was calculated from the frontal plane, and flexion angle was calculated from the sagittal plane. The following is an equation of flexion and lateral flexion of the spinal segments:

(1) $$\cos\varphi =\frac{x_{1}x_{2}+y_{1}y_{2}}{\sqrt{x_{1}^{2}+y_{1}^{2}}\cdot \sqrt{x_{2}^{2}+y_{2}^{2}}}$$

A spinal tilt towards the hand behind the mirror or under the screen is expressed as a positive value. If the tilt was in the opposite direction, it was expressed as a negative value.

In mirror and screen conditions that require bimanual performance, variations in elbow flexion angle were calculated to compare how the angles of each arm change. The acromion, elbow, and wrist markers were connected on each side, and then calculated three-dimensionally to see any elbow angle change during intervention. The following is an equation of elbow flexion angle:

(2) $$\cos\varphi =\frac{x_{1}x_{2}+y_{1}y_{2}+z_{1}z_{2}}{\sqrt{x_{1}^{2}+y_{1}^{2}+z_{1}^{2}}\cdot \sqrt{x_{2}^{2}+y_{2}^{2}+z_{2}^{2}}}$$

Then, the difference between both elbow angles was calculated (seen arm elbow angle–unseen arm elbow angle). If a positive value, the arm which is not the target for the intervention is flexed more, and if a negative value, it is the opposite.

The shoulder angle difference was the measurement of changes in terms of the horizontal plane. It was used to confirm how much the shoulder of the intervention targeted side rotates forward in mirror and screen conditions which require bimanual tasks. By connecting the left acromion and right acromion markers with a straight line, we could verify the change of shoulder position between initial posture and intervention posture. For example, the changing angle based on the left acromion coordinate was calculated when the left upper limb is the target limb (right mirror and left screen conditions). Contrastively, when the right limb is the target (left mirror and right screen conditions), the right acromion was the basis. Equation (1) was used for the calculation. A positive value represents trunk rotation caused by the intervention side coming forward, and a negative value the opposite.

### 2.5. Statistical Analysis

Individual mean values were calculated for the factors of interest. The flexion and lateral flexion of the spinal segments were analyzed via a 2 × 3 (Targeted side (left, right) × device (mirror, screen, movable)) analysis of variance (ANOVA) with repeated measures. The elbow flexion angle difference between both arms and the shoulder angle difference in the horizontal plane were analyzed via a 2 × 2 (Targeted side [left, right] × device (mirror, screen)) analysis of variance (ANOVA) with repeated measures. The threshold for statistical significance was set to *p* < 0.05.

## 3. Results

### 3.1. Flexion of the Spinal Segments

**Figure 2:**
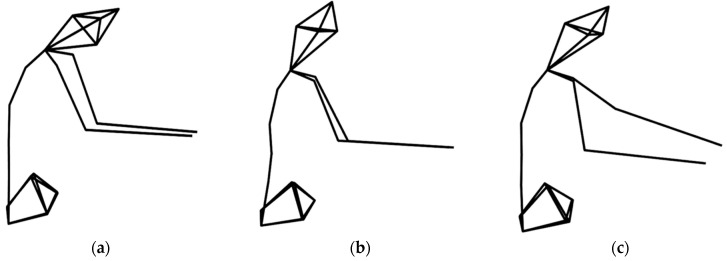
This figure shows spine flexion in each condition. It is the right-side view of the participant during intervention. (**a**) Left mirror condition, (**b**) left screen condition, (**c**) left movable condition.

**Figure 3:**
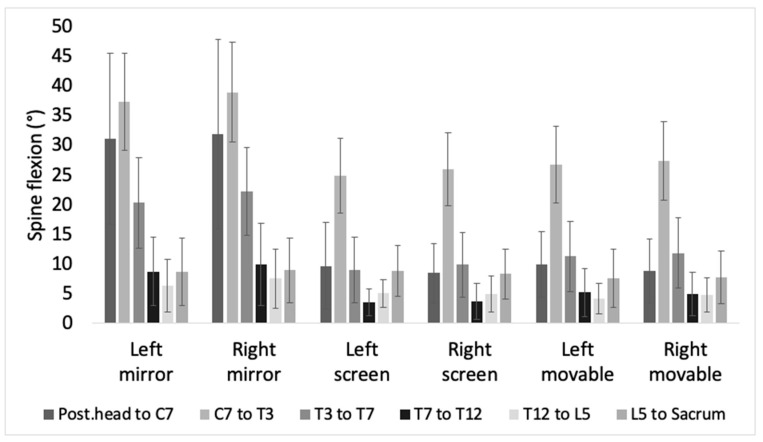
This bar chart shows the mean value of the spine flexion of each segment in each condition.

Figure 2 represents a posture of the spine flexion while using a different device. Figure 3 shows that the average flexion angle while using the screen (left screen mean = 10.18 ± 7.63, right screen mean = 10.22 ± 8.08) and the movable device (left movable mean = 10.85 ± 8.27, right movable mean = 10.95 ± 8.46) were comparable, but it was larger when the mirror (left mirror mean = 18.79 ± 13.11, right mirror mean = 19.93 ± 13.34) was applied. The change of flexion according to device was still significant when comparing by each segment. (Posterior head to c7, F(2,67) = 87.71, *p* < 0.001; C7 to T3, F(2,67) = 140.377, *p* < 0.001; T3 to T7, F(2,67) = 127.664, *p* < 0.001; T7 to T12, F(2,67) = 35.386, *p* < 0.001; T12 to L5, F(2,67) = 13.855, *p* < 0.001; L5 to sacrum, F(2,67) = 4.608, *p* = 0.013).

Pairwise comparison on the device shows a significant difference in flexion between mirror and the other two devices in posterior head to C7, C7 to T3, T3 to T7, T7 to T12, and T12 to L5 (*p* < 0.001). In C7 to T3 (*p* = 0.034), T3 to T7, and T7 to T12 (*p* < 0.001), the difference between screen and movable was additionally found. In L5 to sacrum, there was a significance difference only between screen and movable (*p* = 0.004). 

### 3.2. Lateral Flexion of the Spinal Segments

**Figure 4:**
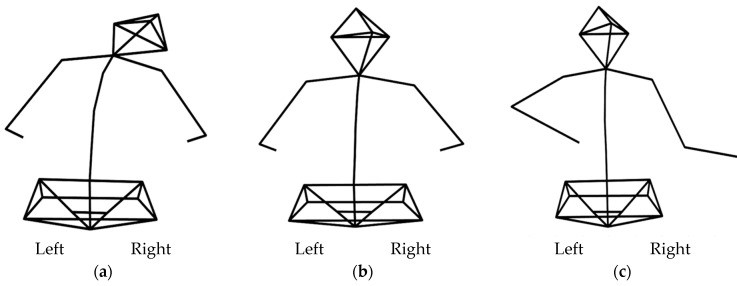
This figure shows spine lateral flexion of each condition. It is the posterior-anterior view of the participant during intervention. (**a**) Left mirror condition, (**b**) left screen condition, (**c**) left movable condition.

**Figure 5:**
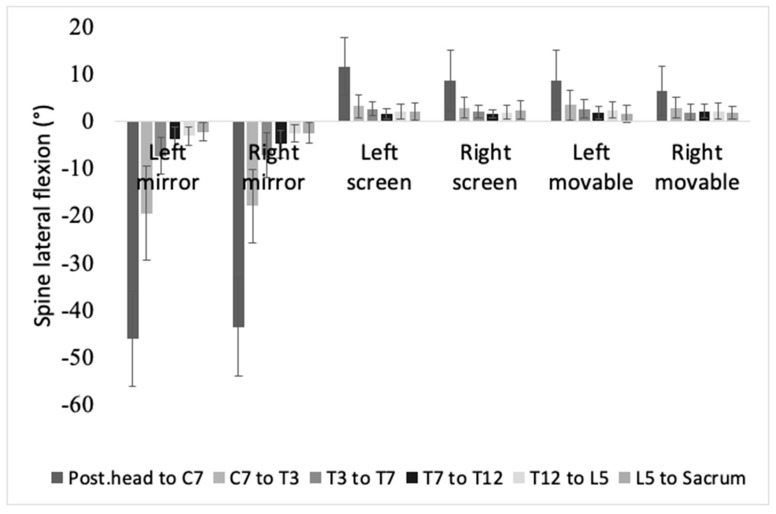
This bar chart shows mean value of spine lateral flexion of each segment in each condition.

Figure 4 is a drawing of how much lateral flexion occurs when using each device. It is the view of the back of the participant in left mirror, left screen, and left movable conditions. In Figure 5, the average lateral flexion angle while using the screen (left screen mean = 3.99 ± 3.84, right screen mean = 3.32 ± 2.67) and the movable device (left movable mean = 3.55 ± 2.62, right movable mean = 3.01 ± 1.77) were comparable. Also, it shows positive value, and depicts the lateral flexion towards the intervention hand. However, in the mirror condition, the tilt was larger, and the direction was away from the target hand (left mirror mean = −13.59 ± 17.17, right mirror mean = −12.97 ± 16.03). 

In all segments, the significant main effect was the device (posterior head to C7, F(2,67) = 1169.219, *p* < 0.001; C7 to T3, F(2,67) = 253.264, *p* < 0.001; T3 to T7, F(2,67) = 273.589, *p* < 0.001; T7 to T12, F(2,67) = 374.696, *p* < 0.001; T12 to L5, F(2,67) = 263.928, *p* < 0.001; L5 to sacrum, F(2,67) = 110.65, *p* < 0.001).

In a pairwise comparison of the device, all segments showed a significant difference between the mirror and the device (mirror vs screen: *p* < 0.001, mirror vs movable: *p* < 0.001). In posterior head to C7, and T7 to T12, a significant difference was shown between screen and movable (*p* < 0.001).

### 3.3. Elbow Flexion Angle Difference between Both Arms

**Figure 6:**
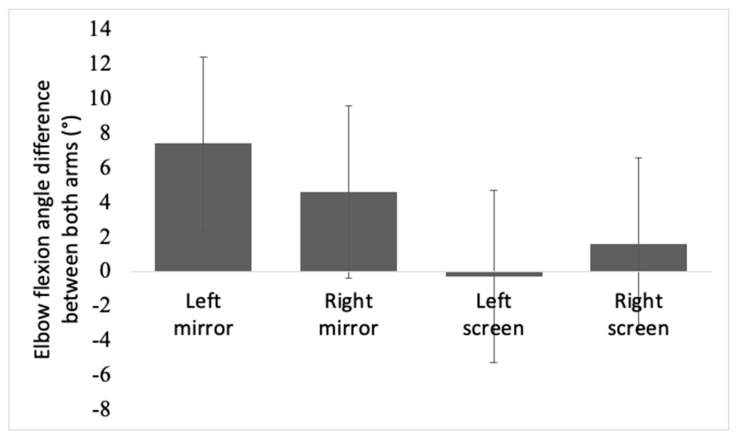
This bar chart shows elbow flexion angle difference between both arms during intervention in mirror and screen conditions. Asterisks represent statistical significance (*p* < 0.05).

Figure 6 is a bar graph that shows the difference between each arm’s elbow flexion angle in mirror and screen conditions. On average, the seen arm made a bigger angle than the unseen arm. Elbow angle difference was bigger in the mirror (mean = 6.06 ± 6.71) than in the screen (mean = 0.64 ± 4.57), and it led to a significant effect between the devices. The difference between F(1,68) = 75.037, *p* < 0.001. The left screen condition and right screen condition values were comparable; however, the right mirror condition showed a bigger angle than the left mirror condition, and showed a significant target side effect, F(1,72) = 5.943, *p* = 0.017. 

### 3.4. Shoulder Angle Difference in the Horizontal Plane

**Figure 7:**
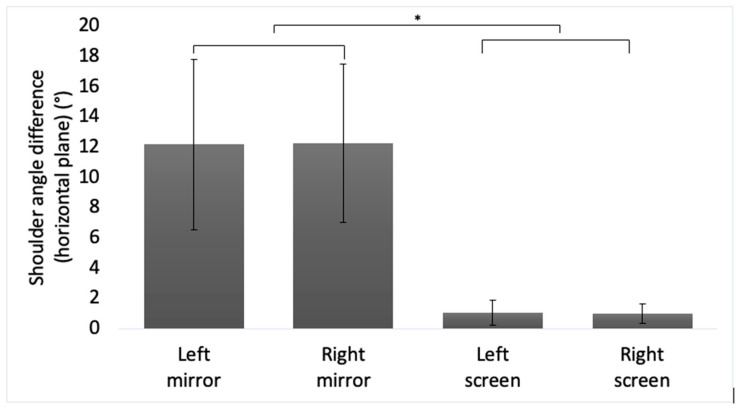
This bar chart shows shoulder angle difference (horizontal plane) during intervention in mirror and screen conditions. Asterisks represent statistical significance (*p* < 0.05).

From Figure 7, it is evident that the intervention-side shoulder makes a forward movement on average. The difference was a mere 1 degree in screen; however, the angle was approximately a 12 degrees rotation forward in mirror. There was a related significant main effect of the device, F(1,68) = 443.784, *p* < 0.001. 

## 4. Discussion

In this experiment, we observed differences between mirror therapy posture and sitting posture that keeps midline. Developed interventions were observed to carry out the task while keeping the posture close to midline sitting. Whereas the mirror condition caused noticeable lateral tilt opposite the target side, screen or movable conditions showed slight lateral tilt toward the target arm. In mirror therapy, postural asymmetry included the shoulder on the side of the intervention coming forward, and different elbow flexion angles between two arms. On the contrary, screen and movable conditions kept the trunk on or close to the midline—conveying minimal changes in shoulder position and elbow flexion angle difference.

Lateral flexion was the most interesting outcome of our experiment. In mirror therapy, one arm was put behind the mirror, thus causing an apparent trunk tilt toward the opposing side. Asymmetric sitting posture was an inevitable step in the preparation to look at the mirror. We prepared a big mirror that comes up to eye level in our experiment setting to prevent going off the midline while sitting [12]. However, lateral tilting was still observed even with bare eyes. 

The size of lateral flexion seems to be related to the participant’s sitting balance. It is not a simple matter of whether one can sit during the performance. Considering whether the participant could keep their tilted posture until the end of the performance was another critical issue. Kim and colleagues [9] reported more significant displacement of center of gravity under the hip in the mirror than in the screen condition. Weight distribution on one side shows the demands of not only the capability in static sitting balance, but also in dynamic sitting balance. The existence of dynamic sitting balance ability is directly related to trunk control ability during task performance. 

Another point of discussion in lateral flexion is the tilting direction. In daily life, manual movements made to perform tasks using hands are created close to the center of mass, or in the same direction as to where the center of mass is headed. In rehabilitation, the same principle is considered. However, during our mirror therapy experiment, the direction of lateral tilt was towards the opposite direction of the target hand, and showed a negative value. In other words, the target limb moved away from the center of mass. 

The size of lateral flexion or directional postural asymmetry may not be an obstacle for healthy participants looking at their movements through the mirror [13]. The change of body segments does not produce a consistent or distinct alteration of the movement sequence. This supports that the central nervous system accounts for appropriate proprioceptive factors in changed posture [14]. 

In the case of stroke patients, the same mirror set can be a challenge. Stroke patients who actively use mirror therapy are hemiparetic or hemiplegic patients. They expect a recovery of affected side movement by utilizing the reflected movement of the healthy side. However, considering the size and direction of the above-mentioned lateral flexion, problems occur. 

It seems essential for the patient to stay seated until the end of the mirror therapy, and to keep the trunk tilted to the unaffected side. If a patient willing to use mirror therapy is in a phase learning midline orientation, it can rather interrupt rehabilitation [15,16,17]. Midline orientation training is weighted in the early stage of stroke rehabilitation [18], and significant improvements in the activities of daily living (ADL) are expected at the same time. Applying mirror therapy to improve hand function may be an optimistic intervention in ADL improvement during this period. However, in the early stage after stroke, tilting the body to the unaffected side for mirror therapy may hinder the midline orientation. On the other side, patients who need education in weight shifting and bearing toward the healthy side may function as another advantage. 

Hiding the affected hand behind the mirror and tilting the trunk towards the unaffected side allows the target hand to move away from the center of mass in intervention. This posture is a unique posture that can only be seen in mirror therapy among rehabilitation interventions [19]. 

The lateral spine flexion led to an asymmetrical angle of both elbow and shoulder [20]. When using the mirror, the elbow angle in front of the mirror was less flexed, and the angle of the arm behind the mirror was more flexed, showing approximately 8 degrees of interlimb elbow angle difference. When performing trunk lateral tilt while putting hands on the table, the angle of the opposite arm of the tilting side is expected to be less flexed. However, interlimb elbow angle difference hints at a strategy to gaze at the mirror while keeping the forearm position placing at the initial posture. Moving the shoulder forward behind the mirror may occur from the effort to reduce the difference in elbow angle of the arms [21]. Considering that mirror therapy is a method to induce bimanual movement, it may be a shoulder movement that a healthy subject makes to keep a similar angle on both elbows. 

The postural change in mirror therapy can trigger various limitations on stroke patients [22,23,24]. It is difficult to expect an equivalent level of healthy beings’ strategy from stroke patients with varying conditions, and there is a high possibility to find postural compensation rather than the above posture. In contrast, the development of mirror therapy by utilizing diverse advanced technology seems to succeed in minimizing postural challenges provoked by intervention posture. The screen condition prevented possible postural challenges caused in the mirror condition for stroke patients. The method that provides the filmed image on the screen placed above the affected side helped keep the midline during the intervention, and aided in keeping the symmetry of both arms’ elbow angle and shoulder level. 

The movable device is a new method that provides a video of the unaffected side over the affected arm. The movable device over the affected limb prevented lateral tilt of trunk, and liberated participants from comparing both arms’ elbow angle and shoulder level [25]. Intervention with the movable condition may also have an advantage as an advanced form of constraint-induced movement therapy (CIMT). Like the traditional CIMT method, the approach could provoke the motion of the “learned-nonuse” of the affected limb, and the visual illusion through the screen may also provide additional advantages [26]. As a movable device, it is expected that the user will get more freedom to perform the task in their preferred position. Although expressed as a movable device in our experiment, this method showed a kinematic reason to develop it into a wearable device using higher technology. A developed type of mirror therapy, and related studies on the weaknesses and strengths of mirror therapy using various devices, are expected in further future studies. 

The postural differences between the three apparatus were analyzed from a kinematic perspective. However, we could not provide an optimal posture and method for each intervention with this single study, and each modern mirror therapy alternative needs feasibility testing. As our interest is in mirror therapy for stroke patients, we expect extensive studies exploring the kinematic benefits in stroke survivors.

## 5. Conclusions

Our study observed the unique posture during mirror therapy. The asymmetric posture while using a mirror may cause challenges in the rehabilitation of stroke patients. However, a mirror therapy concept that uses advanced technology may minimize postural asymmetry, and be an alternative to improve mirror therapy effectiveness. Furthermore, the movable concept may be a method that can provide an additional advantage.

## Data Availability

The data presented in this study are available on request from the corresponding author.
